# Metabolomic Profiling and Mechanotransduction of Single Chondrocytes Encapsulated in Alginate Microgels

**DOI:** 10.3390/cells11050900

**Published:** 2022-03-05

**Authors:** Jacob P. Fredrikson, Priyanka P. Brahmachary, Ayten E. Erdoğan, Zachary K. Archambault, James N. Wilking, Ronald K. June, Connie B. Chang

**Affiliations:** 1 Department of Chemical & Biological Engineering, Montana State University, P.O. Box 173920, Bozeman, MT 59717, USA; jacob.fredrikson@student.montana.edu (J.P.F.); ebruerdogan1995@hotmail.com (A.E.E.); james.wilking@montana.edu (J.N.W.); 2 Department of Mechanical & Industrial Engineering, Montana State University, P.O. Box 173800, Bozeman, MT 59717, USA; pbrahmachary@gmail.com (P.P.B.); archambault.zach@gmail.com (Z.K.A.); 3 Center for Biofilm Engineering, Montana State University, P.O. Box 173980, Bozeman, MT 59717, USA; 4 Department of Microbiology & Cell Biology, Montana State University, P.O. Box 173520, Bozeman, MT 59717, USA

**Keywords:** chondrocytes, microgels, microfluidics, mechanotransduction, osteoarthritis, pericellular matrix, metabolomics

## Abstract

Articular cartilage is comprised of two main components, the extracellular matrix (ECM) and the pericellular matrix (PCM). The PCM helps to protect chondrocytes in the cartilage from mechanical loads, but in patients with osteoarthritis, the PCM is weakened, resulting in increased chondrocyte stress. As chondrocytes are responsible for matrix synthesis and maintenance, it is important to understand how mechanical loads affect the cellular responses of chondrocytes. Many studies have examined chondrocyte responses to *in vitro* mechanical loading by embedding chondrocytes in 3-D hydrogels. However, these experiments are mostly performed in the absence of PCM, which may obscure important responses to mechanotransduction. Here, drop-based microfluidics is used to culture single chondrocytes in alginate microgels for cell-directed PCM synthesis that closely mimics the *in vivo* microenvironment. Chondrocytes formed PCM over 10 days in these single-cell 3-D microenvironments. Mechanotransduction studies were performed, in which single-cell microgels mimicking the cartilage PCM were embedded in high-stiffness agarose. After physiological dynamic compression in a custom-built bioreactor, microgels exhibited distinct metabolomic profiles from both uncompressed and monolayer controls. These results demonstrate the potential of single cell encapsulation in alginate microgels to advance cartilage tissue engineering and basic chondrocyte mechanobiology.

## 1. Introduction

Osteoarthritis (OA) is the most common degenerative joint disease, which affects over 300 million people worldwide [1]. In osteoarthritis, the articular cartilage, the soft load-bearing tissue that lines the interfaces of joints, begins to deteriorate [2]. This is associated with pain and loss of joint function. The tissue in human articular cartilage is spatially homogeneous, including the extracellular matrix (ECM), the territorial matrix, and the pericellular matrix (PCM). The cartilage is primarily comprised of ECM, a hydrated matrix of collagen and proteoglycans [2]. The PCM, which is primarily comprised of type-VI collagen and closely encapsulates the chondrocytes, like a cocoon, directly applies stimuli for chondrocyte mechanotransduction [3]. In normal circumstances, cartilage experiences mechanical loads that vary in frequency and amplitude from activities such as walking, running, and jumping [4]. The PCM protects the chondrocytes from these mechanical loads, but in patients with OA, the PCM is weakened, resulting in increased chondrocyte stress [5]. As chondrocytes are responsible for matrix synthesis and maintenance, it is important to understand how mechanical loads affect the cellular responses of chondrocytes, including their metabolic responses [6].

Many studies have examined the *in vitro* cellular response of chondrocytes to stress by embedding cells in a stiff agarose gel and applying a load to the gel [7,8,9,10]. However, these experiments are mostly performed in the absence of PCM, which may obscure important cellular responses involved in mechanotransduction [3]. Chondrocytes *in vivo* are naturally surrounded by a three-dimensional (3-D) matrix, which has been previously reported to have elastic moduli between 25 and 200 kPa [5,11,12]. Three-dimensional agarose models have achieved similar elastic moduli in the range of 20 to 50 kPa with concentrations of 3–5% *w*/*w* of low-gelling-temperature agarose in phosphate-buffered saline [9]. However, the cells in these models lack a surrounding PCM and may not accurately represent the *in vivo* functions of the PCM. Thus, the cells do not experience the physiologically relevant microenvironments provided by the PCM [3,9].

When cultured in millimeter-scale alginate beads, chondrocytes exhibit phenotypes more similar to those in *in vivo* conditions [13,14,15]. These phenotypes include increased glycosaminoglycan (GAG) and collagen synthesis, both of which are components of both the PCM and ECM [14]. In alginate, chondrocytes synthesize a PCM, yet the elastic moduli of alginate (~1 kPa) cannot reach the elastic moduli of the PCM, which can more accurately depict *in vivo* mechanical loads in high-stiffness agarose [16,17,18,19]. One method of retaining the PCM around the cells is to first grow the cells in a 3-D matrix of alginate and then use various digestive processes to isolate the cells [14]. However, this second digestion may have adverse effects on chondrocytes.

Therefore, there remains a need for a technique in which cells can undergo PCM formation in alginate without secondary digestion prior to constructing a bulk 3-D matrix. With such a technique, cells can be embedded into a stiffer agarose matrix while retaining properties that better mimic the *in vivo* microenvironment. One such method is the use of drop-based microfluidics to encapsulate single cells in microscale hydrogels. This approach utilizes fluid flow through microchannels to create monodisperse picoliter-sized microgel droplets. By flow-focusing an inner alginate hydrogel precursor with an outer oil phase, alginate microgels can be fabricated at kilohertz rates [19]. This method has been used to encapsulate numerous mammalian cell types [19,20]. These microgels provide nearly homogenous, highly tunable, 3-D growth environments for cells. Compared to bulk hydrogels, microgels have fewer nutrient limitations and favorable nutrient transfer characteristics due to the high surface area to volume ratio and small length scale [21]. The individual microgels are easily manipulated through pipetting and centrifugation, allowing for the facile transfer of encapsulated cells between growth environments.

Here, we demonstrate that drop-based microfluidics enables the culture of single chondrocytes in alginate microgels for cell-directed PCM synthesis that closely mimics the *in vivo* microenvironment. When encapsulated into a stiff agarose construct, these microgel-encapsulated chondrocytes create a cartilage model that includes components of both the PCM in the collagen VI, which surrounds the cells, and ECM in the stiff agarose, providing a more physiologically relevant compression model. Chondrocytes form PCM over 10 days in these single-cell microenvironments in comparison to cells grown in a monolayer environment, which do not form PCM. We investigated the effects of cyclical dynamic compression on cells cultured in microgels and as monolayers by encapsulating single-cell microgels and monolayer controls in high-stiffness agarose to mimic the cartilage PCM. After physiological dynamic compression, microgels exhibited distinct metabolomic profiles from both uncompressed and monolayer controls [8]. These microgel-encapsulated chondrocytes developed PCM similar to that of the endogenous PCM and have distinct metabolomic profiles compared to monolayer controls. Upon metabolomic pathway analysis, microgel-encapsulated chondrocytes have up-regulated pathways related to amino acid synthesis and central energy metabolism. These results demonstrate the potential of single-cell encapsulation in alginate microgels to advance cartilage tissue engineering and basic chondrocyte mechanobiology through single-cell matrix formation studies and more realistic cartilage models.

## 2. Methods

### 2.1. Chondrocyte Harvest and Culture

Human Primary Chondrocytes (HPC) were obtained from *n* = 3 Stage-IV osteoarthritis patients undergoing total joint replacement under IRB approval using established methods [22,23]. Cells were isolated by digestion with Collagenase Type I (2 mg/mL) (Gibco, Waltham, MA, USA) for 14 h at 37 °C. Isolated chondrocytes were cultured for 10 days in Dulbecco’s Modified Eagle’s medium (DMEM) (Gibco, Waltham, MA, USA) supplemented with Fetal Bovine Serum (FBS) (10% *v*/*v*) (Bio-Techne, Minneapolis, MN, USA), penicillin (10,000 I.U./mL), and streptomycin (10,000 μg/mL) (Sigma, St. Louis, MO, USA) (hereafter referred to as complete media) in 5% CO_2_ at 37 °C. Chondrocytes were passaged at 90% confluency and seeded at a density of 1 × 10^5^ cells onto 25 × 25 mm microscope coverslips (Fisherbrand, Hampton, NH, USA). The coverslips with the attached cells were placed in a 60 × 15 mm tissue culture dish containing complete media supplemented with L-sodium ascorbate (50 μg/mL) (Sigma, St. Louis, MO, USA) and incubated for 10 days. The media were exchanged every other day. Monolayers grown in complete media lacking L-sodium ascorbate for 10 days at 37 °C were used as controls. Cells were imaged on days 0, 5, and 10 for collagen VI production. Similarly, SW1353 chondrocytes, a cell line initiated from a human chondrosarcoma and obtained from the American Type Culture Collection (ATCC, Manassas, VA, USA), were seeded on microscope coverslips at a density of 1 × 10^5^ cells and cultured in complete media supplemented with L-sodium ascorbate (50 μg/mL) for 10 days in 5% CO_2_ at 37 °C and imaged on days 0, 5, and 10. Monolayers grown in complete media lacking L-sodium ascorbate for 10 days at 37 °C were used as controls. SW1353 cells were used between passages 5 and 20, and HPCs were used between passages 2 and 5 post-harvest.

### 2.2. PDMS Microfluidic Device Fabrication

Polydimethylsiloxane (PDMS) microfluidic devices were fabricated using standard soft lithography techniques [24]. Negative master molds were fabricated on 3-inch-diameter silicon wafers (University Wafer Inc., Boston, MA, USA University Wafer ID: 447) using UV crosslinked Nano SU-8-100 photoresist (Microchem, Round Rock, TX, USA) patterned with photomasks printed on high-resolution transparent plastic film (CAD/Art Inc., Bandon, OR, USA). Two-component Sylgard 184 PDMS (Dow Chemical, Midland, MI, USA) was mixed at a 10:1 ratio by mass, poured over wafers in a 100 mm petri dish (Fisherbrand, Hampton, NH, USA), and degassed. The devices were baked at 55 °C for at least 4 h. The cured devices were cut from the master and ports were punched using a 0.75 mm ID biopsy punch (Well Tech, Taichung, Taiwan). The devices were plasma-bonded to 3 × 2 in glass slides (VWR, Randor, PA, USA) for drop makers and 25 × 25 mm type-0 coverslips (VWR, Randor, PA, USA) for imaging arrays by exposing the PDMS and glass to oxygen plasma for 1 min at high power, 45 watts, and 700 mTorr oxygen pressure using a plasma cleaner (Harrick Scientific Corp, Pleasantville, NY, USA, PDC-002). The bonded devices were baked at 55 °C for at least 1 h to increase the strength of the plasma bond. Following baking, drop makers were filled with a hydrophobic treatment (PPG Industries, Pittsburgh, PA, USA Aquapel) for 5 min, flushed with air, and baked at 55 °C again for 1 h.

### 2.3. Preparation of Precursor Solutions

#### 2.3.1. Oil Preparation

The surfactant Krytox 157 FSH (4% *w*/*w*) (Miller-Stephenson, Danbury, CT, USA) was added to the fluorinated oil, HFE 7500 (3M, Saint Paul, MN, USA 3M Novec). The solution was filtered through a 0.2 µm hydrophobic polytetrafluoroethylene (PTFE) syringe filter (GVS, Bloomer, WI, USA).

#### 2.3.2. Alginate Precursor Preparation (A)

CaCl_2_ (80 mM) (ACROS Organics, Waltham, MA, USA), Na_2_EDTA (Ethylenediaminetetraacetic acid) (80 mM) (Fisher Chemical, Hampton, NH, USA), and 3-(Morpholin-4-yl)propane-1-sulfonic acid (MOPS) (40 mM) (VWR, Randor, PA, USA) were mixed with ultra-pure water (50 mL) (18.2 MΩ●cm, MilliQ) and the pH was adjusted to 7.2 using NaOH (2 M) (VWR, Randor, PA, USA). Sodium alginate (1.5% *w*/*w*) (Sigma, St. Louis, MO, USA, 9005-38-3) was added to the solution (10 mL) and the solution was filtered through a 0.2 µm hydrophilic polyethersulfone (PES) syringe filter (MilliporeSigma Millex, Burlington, MA, USA).

#### 2.3.3. Alginate Precursor Preparation (B)

Zn(CH_3_CO_2_)_2_ (80 mM) (Alfa Aesar, Haverhill, MA, USA), EDDA (Ethylenediaminediacetic acid) (80 mM) (TCI Chemicals, Tokyo, Japan), and MOPS (40 mM) were mixed with ultra-pure water (50 mL) (18.2 MΩ●cm, MilliQ) and the pH was adjusted to 7.2 using NaOH (2 M). Sodium alginate (1.5% *w*/*w*) was added to the solution (10 mL) and the solution was filtered through a 0.2 µm PES syringe filter.

### 2.4. Cell Encapsulation in Microgels

To prepare cells for encapsulation, the cells were removed from monolayers using Trypsin-EDTA (2 mL) and suspended in media. The cells were centrifuged at 500× *g* for 5 min (Thermo Scientific, Waltham, MA, USA ST-16) and washed with phosphate-buffered saline (PBS) (1X, 10 mL) twice. In order to minimize the number of empty drops, the cells were then suspended in alginate precursor solution ‘A’ at 5 × 10^6^ cells/mL (final concentration: 2.5 × 10^6^ cells/mL) and loaded into 1 or 3 mL syringes (BD, Franklin Lakes, NJ, USA, Luer lock). Following previous protocols, the cells were encapsulated in 100 µm-diameter alginate microgels [19]. The continuous flow rate used was *Q*_Oil_ = 2000 µL/h and the dispersed phase flow rate used was *Q*_AlgA_ = *Q*_AlgB_ = 250 µL/h. Microgels were produced in parallel by running two drop-making devices for 4 h to encapsulate at least 1 × 10^7^ cells. After encapsulation and gelation, the microgels were washed with HFE 7500 (500 µL) and 1H,1H,2H,2H-Perfluoro-octanal (PFO) (20% *v*/*v*) (VWR, Randor, PA, USA) in HFE 7500 (100 µL) for each 125 µL of microgel 2–3 times to remove the surfactant. The microgels were collected from the oil phase with PBS (1X, 500 µL) and suspended in appropriate media (see the following section).

### 2.5. Cell Culture in Microgels

Approximately 200 µL of microgels containing ~1.3 cells/microgel were placed in each well of a six-well plate (Falcon Corning, Corning, NY, USA) and covered with media (2 mL). The cells were either cultured with complete media or complete media supplemented with L-sodium ascorbate (50 µg/mL) to promote collagen synthesis. Every 3 days, the media were exchanged by collecting the contents of each well, centrifuging at 500× *g* for 2 min, removing the supernatant, and suspending the microgels in new media (2 mL).

### 2.6. Cell Viability Assay

After 10 days of culture, microgel-encapsulated SW1353 cells (100 µL) were mixed with PBS (1X, 800 µL) and Trypan Blue (100 µL) (Corning, Corning, NY, USA). An amount of 10 µL of the solution was pipetted onto a hemocytometer (Reichert Depew, Buffalo, NY, USA) to quantify live and dead cells. Each measurement contained 10–40 cells, for a total of approximately 260–280 cells for both microgels with and without ascorbate.

### 2.7. Fluorescence Imaging and Analysis

SW1353 cells were imaged using an epi-fluorescent microscope (Leica, Wetzlar, Germany, DMi-8) with a 40X objective over 10 days to capture the brightfield and auto-fluorescence (ex. 350 nm/em. 470 nm). For autofluorescence imaging, each day, 30 µL of microgels and media were flowed into a microfluidic chamber with a height greater than the microgel diameter. Approximately 20 cells per growth condition, i.e., monolayers and microgels with or without ascorbate, were imaged. The background intensity was subtracted from each image using Fiji ImageJ and the fluorescence intensity of each cell was measured using the Fiji ImageJ distribution of TrackMate [25,26].

### 2.8. Immunofluorescence Staining and Confocal Imaging

#### 2.8.1. Monolayer Controls

HPCs and SW1353 cells grown in monolayers on microscope coverslips were fixed in paraformaldehyde (4% *v*/*v*)–PBS (1X) for 10 min at room temperature, followed by three 5 min washes with PBS (1X). The cells were permeabilized with a blocking solution containing Triton X-100 (0.1% *v*/*v*)–Bovine Serum Albumin (1% *v*/*v*)–PBS (1X) for 30 min at room temperature. The cells were then incubated with the primary antibody to collagen VI (Rabbit Polyclonal Anti-collagen VI antibody, ab6588 from Abcam, Boston, MA, USA) in Triton X-100 (0.1% *v*/*v*)–Bovine Serum Albumin (1% *v*/*v*)–PBS (1X) for 1 h at room temperature. After three 5 min washes with PBS (1X), the cells were incubated with a mixture of the secondary antibody Donkey anti-Rabbit IgG H&L Alexa Fluor^®^488 (Abcam, Boston, MA, USA) and Vibrant^TM^DyeCycle^TM^ Violet Stain for Nuclei (Invitrogen, Waltham, MA, USA) in Triton X-100 (0.1% *v*/*v*)–Bovine Serum Albumin (1% *v*/*v*)–PBS (1X) for 1 h at room temperature. The cells were washed 3 times with PBS (1X) and the coverslip was mounted on a glass slide with ProLong^TM^ Diamond Antifade Mountant (Invitrogen, Waltham, MA, USA). Digital images were acquired on a Leica TCS SP8 confocal microscope with the Leica Application Suite Advanced Fluorescence software.

#### 2.8.2. Microgel-Encapsulated Chondrocytes

Alginate beads with encapsulated chondrocytes were spun down at 500× *g* for 2 min, washed with PBS (1X), and then fixed with paraformaldehyde (4% *v*/*v*)–PBS (1X) for 15 min at room temperature. The paraformaldehyde-fixed microgel-encapsulated cells were permeabilized with a blocking solution containing Triton X-100 (1.0% *v*/*v*)–Bovine Serum Albumin (3% *v*/*v*)–PBS (1X) for 30 min at room temperature. The microgels were then incubated with the primary antibody to collagen VI (Rabbit Polyclonal Anti-collagen VI antibody, ab6588 from Abcam, Boston, MA, USA) in Triton X-100 (1.0% *v*/*v* )–Bovine Serum Albumin (3% *v*/*v*)–PBS (1X) for 1 h at room temperature. Following three washes of 5 min each with PBS (1X), the encapsulated samples were incubated with a mixture of secondary antibody Donkey anti-Rabbit IgG H&L Alexa Fluor^R^488 (Abcam, Boston, MA, USA) and Vibrant^TM^DyeCycle^TM^ Violet Stain for Nuclei (Invitrogen, Waltham, MA, USA) in Triton X-100 (1.0% *v*/*v* )–Bovine Serum Albumin (3% *v*/*v*)–PBS (1X) for 1 h at room temperature. The microgel constructs were washed three times with PBS (1X). An amount of 100 μL of the alginate beads was cytospun onto single frosted adhesive slides (Tanner Scientific, Sarasota, FL, USA) using a Thermo Scientific Cytospin^TM^ 4 Cytocentrifuge for immunocytochemistry and mounted with ProLong^TM^ Diamond Antifade Mountant (Invitrogen, Waltham, MA, USA).

### 2.9. Encapsulation in High-Stiffness Agarose, Mechanical Stimulation, and Metabolite Extraction

After 9 days in culture, microgels and SW1353 cells from each condition were mixed with low-melting-temperature agarose (Sigma Aldrich, St. Louis, MO, USA) at a final agarose concentration of 4.5% *w*/*w* [9,27]. The final cell concentrations for each condition were 1 × 10^6^ cells/mL, with each gel being a 0.5 mL cylinder of 12.75 mm in height. The agarose was allowed to cool to room temperature to form a gel. The agarose gels were each placed in individual wells of a 12-well plate (Falcon Corning, Corning, NY, USA) and cultured for 2 days under appropriate experimental conditions. After 2 days, each gel was loaded onto a custom-built bioreactor at 37 °C, 5% CO_2,_ and 95% relative humidity in PBS and preloaded at 5% strain [8]. After 30 min, 5% ± 2% cyclic strain at 1.1 Hz was applied for 15 min. The gels were washed with PBS and frozen for 1 min with liquid nitrogen. The gels were then crushed and placed in a −80 °C freezer for 1 h. The gels were removed from the freezer and a 1 mL solution of equal volumes of HPLC-grade methanol (Fisher Chemical, Hampton, NH, USA) and acetone (Sigma Aldrich, St. Louis, MO, USA) was added to each sample. The samples were vortexed every 5 min for 20 min and kept at −20 °C overnight. The next day, the samples were centrifuged at 13,000 rpm for 10 min at 4 °C (Thermo Scientific, Waltham, MA, USA Sorvall Legend X1R). The supernatant was removed and placed in a speed-VAC (Savant SC110) for 6.5 h. The resulting pellet was suspended in a 1:1 mixture of HPLC-grade water (Fisher Chemical) and acetonitrile (Fisher Chemical, Hampton, NH, USA) and placed in a −80 °C freezer until used for HPLC–MS.

### 2.10. Untargeted Metabolic Profiling

The changes in cellular activity were studied using untargeted metabolomic profiling by HPLC–MS (high-performance liquid chromatography coupled to mass spectrometry). Chromatography was performed in the normal phase with established protocols using a Cogent Diamond Hydride HILIC 150 × 2.1 mm column in an Agilent 1290 UPLC system [28]. Mass spectrometry was performed using an Agilent 6538 Q-TOF. Metabolites with a median intensity of zero across all experimental groups were excluded from the analysis. Undetected remaining metabolites (i.e., intensity of zero) were replaced with a value of one half of the minimum peak intensity for statistical analyses. Statistical analysis was performed in Metaboanalyst. The data were first log-transformed and standardized. The metabolomic profiles between the experimental groups were compared using principal components analysis (PCA), hierarchical clustering, volcano plot analysis, and pathway enrichment analysis. Significance was assessed with false discovery rate corrections using a significance level of 0.05 selected *a priori*.

### 2.11. Rheology

The storage and loss moduli of each gel used were measured using a TA Instruments AR-G2 parallel plate rheometer. The gel mixtures were poured into 2 mL molds and gelled. They were then loaded onto the rheometer and 1% strain was applied from 100 to 0.1 rad/s.

### 2.12. Statistical Analysis

The SW1353 cell viabilities were compared using a Welch Two-Sample *t*-test in R [29,30]. The rate of autofluorescence increase was calculated using a weighted multiple linear regression in R [29,30]. Each measurement was weighted by the inverse variance of the mean of each sample.

## 3. Results and Discussion

### 3.1. Chondrocyte Encapsulation in Alginate Microgels and Dynamic Compression Workflow

**Figure 1:**
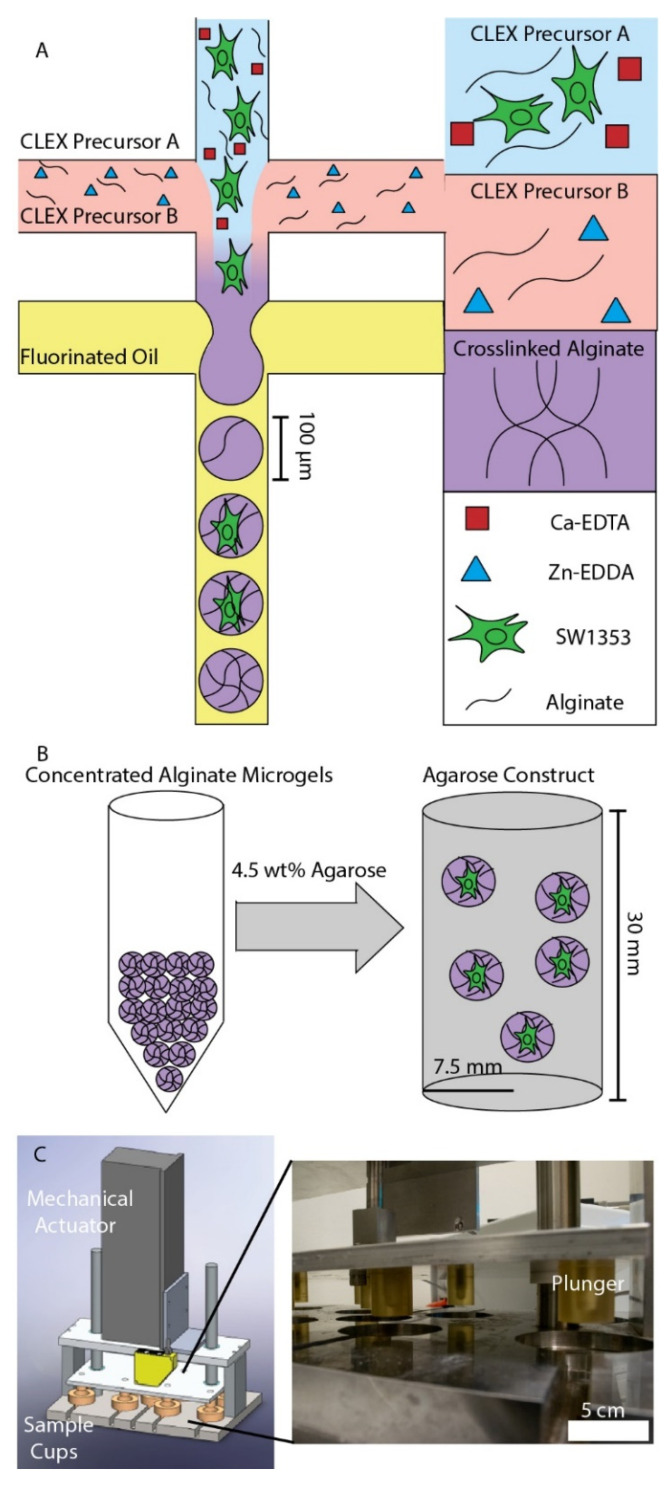
Experimental workflow: (**A**) Schematic of alginate microgel production. Cell-laden CLEX precursor A was mixed with CLEX precursor B and flow focused with 4% FSH surfactant in HFE 7500 oil. (**B**) After 9 days of culture, concentrated alginate microgels were mixed with agarose to a final agarose concentration of 4.5% *w*/*w* and 80% *v*/*v*. (**C**) After 2 days, a custom-built bioreactor applied 5 ± 2% cyclic strain to the agarose constructs containing alginate microgels. Following cyclic compression, metabolites were extracted from the samples and analyzed using HPLC–MS. In the close-up image (**C** right), the sample cups were removed to visualize the plungers.

Previous studies investigated the effects of alginate macrogels on the formation of the pericellular matrix (PCM), but, while these macrogels support the formation of PCM, they have insufficient stiffness to mimic the *in vivo* elastic modulus surrounding chondrocytes, which is between 25 and 200 kPa [13,14,15]. Here, drop-based microfluidics was used to encapsulate single cells (SW1353 and HPC) in alginate microgels (Figure 1A). To produce alginate microgels in a biocompatible manner, the competitive ligand exchange crosslinking (CLEX) method for crosslinking alginate was used [19]. Briefly, 1.5% *w*/*w* alginate was mixed with two solutions of 80 mM Ca-EDTA and Zn-EDDA. Upon mixing the two precursor solutions, Zn^2+^ binds to EDTA, releasing Ca^2+^, which crosslinks the alginate. After 9 days in culture, alginate microgels were concentrated via centrifugation, suspended in 4.5% *w*/*w* agarose, and gelled (Figure 1B). The agarose constructs were allowed to equilibrate over two days to ensure that the process of creating the agarose constructs did not affect the metabolic activity of the cells [9]. The agarose constructs were then placed in a custom-built bioreactor and then subjected to physiological dynamic compression of 5 ± 2% cyclic strain at 1.1 Hz for 15 min (Figure 1C) [8]. Following compression, metabolites were extracted from the chondrocytes and analyzed using HPLC–MS.

### 3.2. Immunofluorescence Staining of PCM Formation in Alginate Microgels

**Figure 2:**
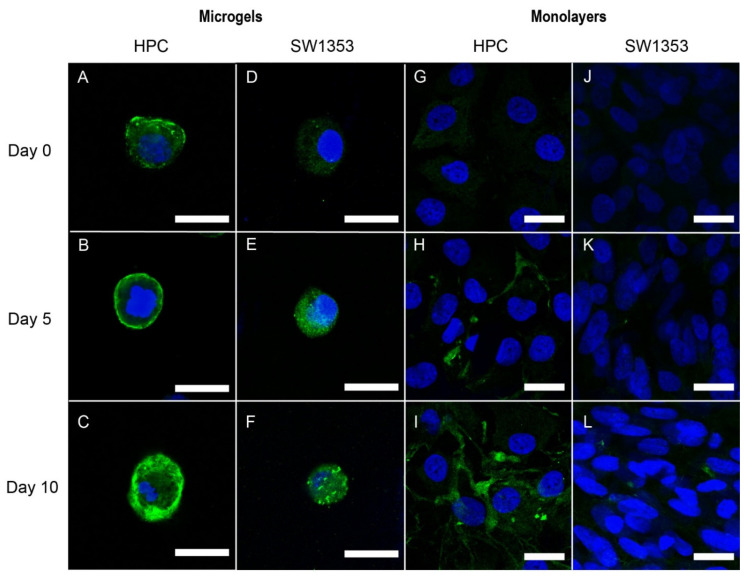
Human Primary Chondrocyte (HPC) (**A**–**C**,**G**–**I**) and SW1353 cells (**D**–**F**,**J**–**L**) in 1.5% *w*/*w* alginate microgels and monolayers cultured with 50 µg/mL ascorbate. Cells were stained for collagen VI (green) and nuclei (blue). Cells were imaged on days 0, 5, and 10. Scale bars are 20 µm.

To visualize the PCM formation, we compared two different cell lines, the SW1353 human chondrosarcoma cell line and human primary chondrocytes harvested from osteoarthritic human knees. The cells were fixed on days 0, 5, and 10 and stained using polyclonal anti-collagen VI antibodies for the primary component of the PCM, collagen VI. Confocal microscopy was used to image collagen VI formation surrounding the single cells. When cultured with ascorbate, which is known to increase collagen production [31], the HPC cells encapsulated in 3-D microgels produced more collagen VI compared to the HPCs cultured in monolayers, SW153 cells encapsulated in microgels, and SW153 cells encapsulated as monolayers (Figure 2). The HPC cells produced collagen VI that closely surrounded the outside of the cell. A thin, approximately 1 µm, layer of collagen VI formed around most of the HPC cells in microgels by day 5 (Figure 2B), and by day 10, the collagen VI layer ranged from 2 to 5 µm thick, surrounding the entire perimeter of the cell (Figure 2C). Similarly, the SW1353 cells cultured in microgels showed collagen VI staining by day 5. By day 10, the majority of the cell was covered with collagen VI when ascorbate was present in the media (Figure 2D–F). However, the SW1353 cells produced less collagen VI than the HPC cells, and the resulting collagen VI had a less cocoon-like appearance. When cultured without ascorbate, the HPC and SW1353 cells showed similar trends, with more collagen VI production in the microgels than the monolayers, but less collagen VI production than when cultured with ascorbate (data not shown).

### 3.3. Immunofluorescence Staining of Pericellular Matrix Formation in Monolayers

As a control study, we verified that the formation of PCM in typical two-dimensional (2-D) cellular monolayers was undetectable under these conditions [32]. HPC cells cultured in 2-D monolayers with ascorbate produced a small amount of collagen VI (Figure 2G–I). While the HPC cells cultured in monolayers produced collagen VI, this collagen VI was typically located in small, concentrated regions and did not surround the cells compared to the cells cultured in microgels or naïve cells in the endogenous PCM (Figure 2I) [3]. In contrast, the SW1353 cells cultured in 2-D monolayers showed no collagen VI on day 0 and had negligible collagen VI staining by day 10 when ascorbate was present in the media, indicating a lack of PCM formation (Figure 2J–L). HPC and SW1353 cells cultured as monolayers without ascorbate showed low levels of collagen VI compared to when cultured with ascorbate (data not shown).

### 3.4. Auto-Fluorescence of PCM Formation in Alginate Microgels

**Figure 3:**
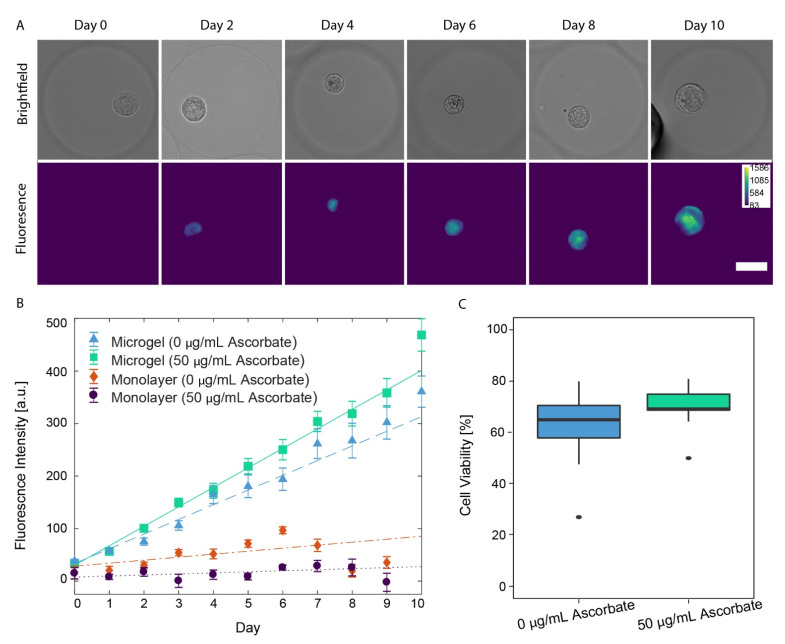
(**A**) Brightfield and fluorescence images (ex. 350/em. 470) of SW1353 cells encapsulated in alginate microgels and cultured with 50 µg/mL ascorbate. Scale bar is 25 µm. (**B**) Measured mean fluorescence intensities of imaged SW 1353 cells plotted with a weighted multiple linear regression. SW1353 cells encapsulated in microgels cultured without ascorbate (blue triangle, dashed), encapsulated in microgels cultured with ascorbate (green square, solid), monolayers cultured without ascorbate (orange diamond, dashed–dotted), and monolayers cultured with ascorbate (purple circle, dotted). (**C**) Ten-day cell viability for SW1353 cells encapsulated in alginate microgels. Cell viability between cells cultured with or without ascorbate in microgels did not vary (*p*-value = 0.0957).

In addition to immunofluorescence staining, we quantified collagen formation by measuring the auto-fluorescence signal produced from SW1353 cells under epi-fluorescence imaging. Collagen, a main component of the PCM, shows substantial auto-fluorescence upon excitation by light ranging from UV to blue (365–415 nm) [33,34,35]. Although collagen VI is a specific marker of chondrocyte PCM formation, other types of collagen (I, II, and IV) can also auto-fluoresce, but we are not able to distinguish types of collagen using this method [3,33,34,35]. Therefore, this label-free approach enables general collagen-based PCM formation to be quantified over time. Only SW1353 cells were studied, as they are more readily available compared to the human primary chondrocytes. Each day over 10 days, SW1353 cells grown in microgels and monolayers with and without ascorbate were imaged for auto-fluorescence. Similar to immunofluorescence staining, the SW1353 cells cultured in microgels produced the most collagen. We show representative images of single cells grown in alginate microgels, which display an increase in the auto-fluorescence of the PCM over time (Figure 3A). The auto-fluorescence signal closely surrounding the cell is similar to that of endogenous PCM, where collagen VI surrounds the cell in a cocoon-like manner [3]. As a control experiment, we verified that when SW1353 cells were cultured in a monolayer with and without ascorbate, there was not a noticeable auto-fluorescence signal, indicating the absence of collagen (Figure S1A,B).

As another control experiment, we verified that, when ascorbate was not present in the media, there was a decrease in the rate of change in the PCM auto-fluorescence signal compared to that when ascorbate was present. Representative images of SW1353 cells grown in microgels without ascorbate still showed an increase in the auto-fluorescence signal over time (Figure S1C); however, upon quantitative image analysis, a significant decrease in the rate of auto-fluorescence signal change was observed compared to that of SW1353 cells grown in microgels with ascorbate. When SW1353 cells were cultured in microgels with ascorbate, the auto-fluorescence signal increased by 37.1 ± 2.4 a.u./day (Figure 3B, green squares). In contrast, when SW1353 cells were cultured in microgels without ascorbate, the auto-fluorescence signal increased by 28.0 ± 2.8 a.u./day (Figure 3B, blue triangles), a decrease of 9.1 ± 3.6 a.u./day (*p*-value = 6.91 × 10^−7^). The control monolayer SW1353 cells, when cultured with ascorbate, showed negligible increases in the auto-fluorescence signal over time as the signal increased 2.0 ± 2.0 a.u./day (Figure 3B, purple circles). This is a 35.1 ± 3.0 a.u./day decrease compared to that of SW1353 cells cultured in microgels with ascorbate (*p* < 2 × 10^−16^). The presence of ascorbate did not significantly change the rate of auto-fluorescence increase in the monolayers (*p* < 0.016, Figure 3B, orange diamonds). There was no statistically significant difference between the monolayers cultured with or without ascorbate.

Overall, collagen production in the PCM showed similar trends, with both immunofluorescence and auto-fluorescence. Collagen production increased in microgels compared to monolayers, both with and without ascorbate present. These data indicate that the single-cell encapsulation of chondrocytes within alginate microgels, regardless of ascorbate, dramatically increases collagen production compared to monolayer controls.

### 3.5. Chondrocyte Viability in Alginate Microgels

After 10 days of culture in alginate microgels, SW1353 cell viability was evaluated using a dead stain (Trypan Blue exclusion). When 50 µg/mL of ascorbate was present in the media, the cell viability was 70.3 ± 4.3%. Without ascorbate, the cell viability was 62.4 ± 7.7% (Figure 3C). The difference in cell viability was not significant with and without ascorbate in the media (*p*-value = 0.0957, Welch two-sample *t*-test), indicating that 50 µg/mL of ascorbate did not affect SW1353 viability in alginate microgels.

### 3.6. Elastic Moduli Characterization of Hydrogel Constructs

To create agarose–alginate constructs, 1.5% *w*/*w* alginate microgels were suspended in a 4.5% *w*/*w* agarose solution, poured into a cylindrical mold, and gelled. To quantify the mechanical properties of these hydrogels with and without embedded microgels, the storage and loss moduli of the 1.5% *w*/*w* alginate, the 4.5% *w*/*w* agarose, and the 4.5% *w*/*w* agarose with 20% *v*/*v* 1.5% *w*/*w* alginate microgels were measured on a parallel plate rheometer (Table S1). The 1.5% *w*/*w* alginate (*G’*~660 Pa, *G”*~35 Pa, *ω* = 1 rad/s) had storage and elastic moduli two orders of magnitude lower than those of the agarose (*G’*~32 kPa, *G”*~2.3 kPa, *ω* = 1 rad/s) (Figure S2). There was no significant difference between the stiffnesses of the agarose gels with and without embedded alginate microgels, indicating that the bulk mechanical properties were not affected by microgel encapsulation.

### 3.7. Metabolomic Profiling of Hydrogel Constructs Containing Compressed and Uncompressed Monolayer and Microgel Cells

**Figure 4:**
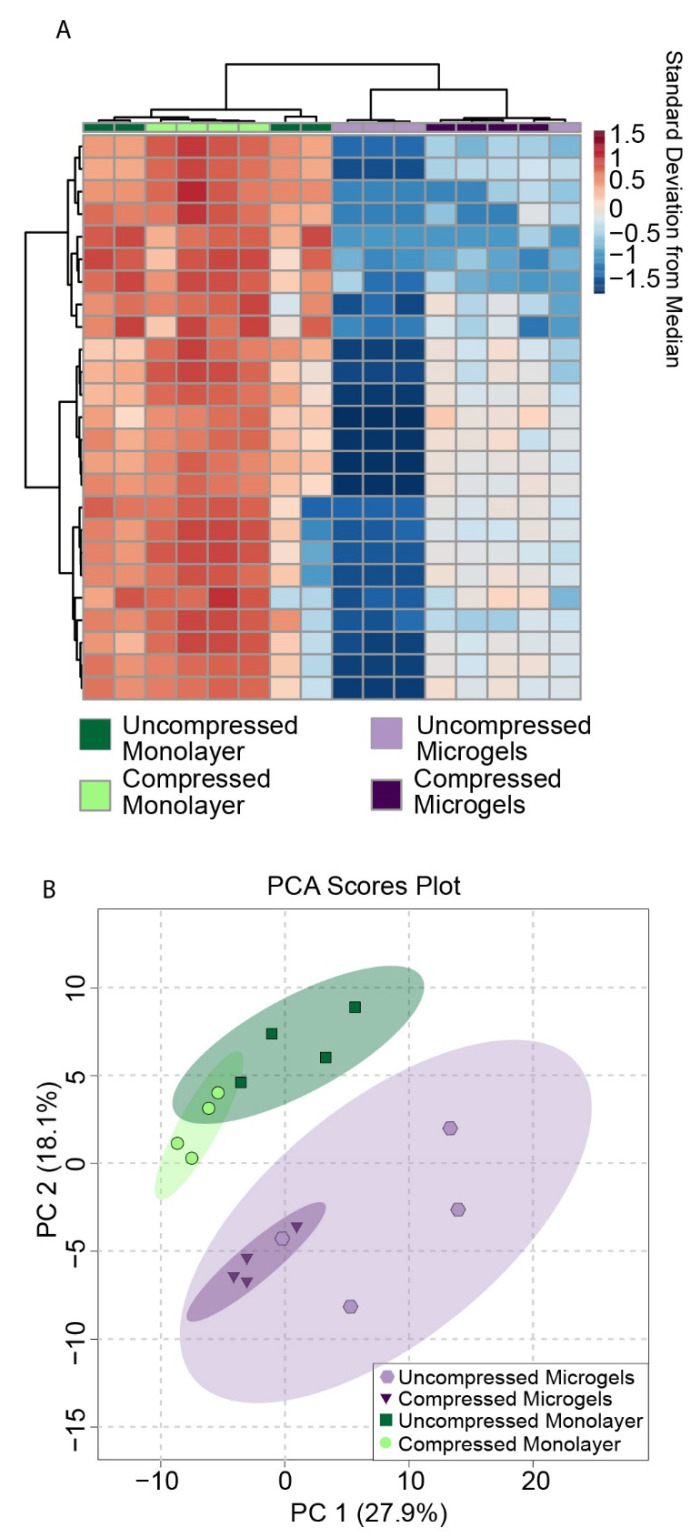
(**A**) Heat map of top 25 metabolites from SW1353 cells. (**B**) Two-dimensional score plot of PCA analysis for metabolites from SW1353 cells cultured with complete media supplemented with 50 μg/mL L-sodium ascorbate.

**Figure 5:**
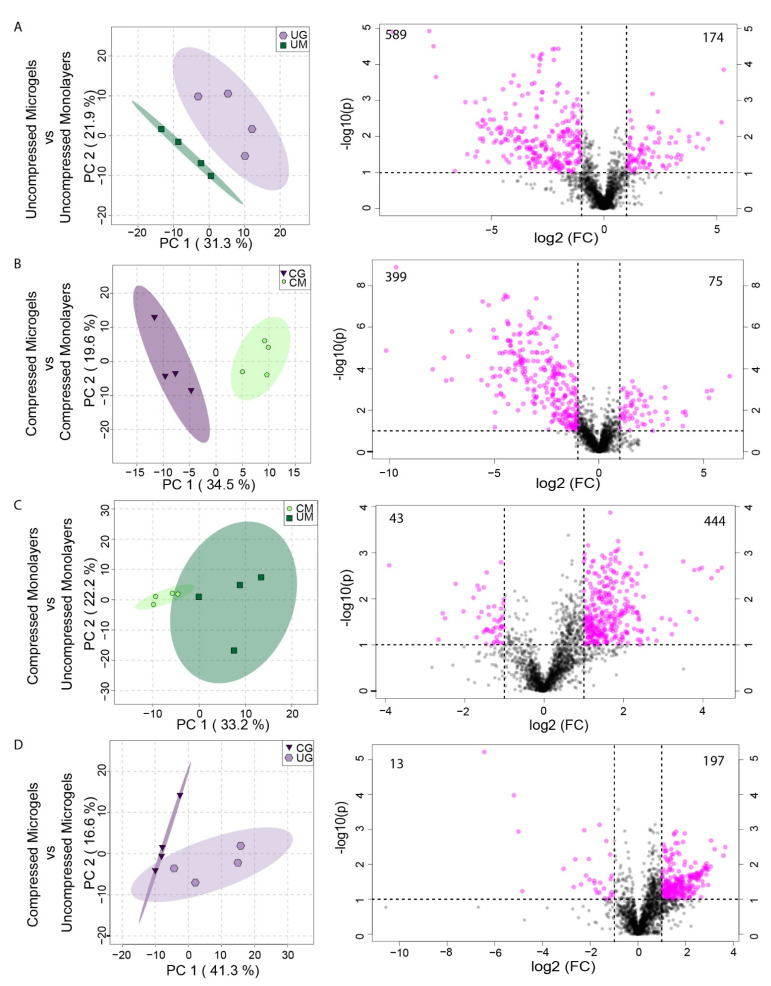
Two-dimensional score plots for PCA analysis (left) and volcano plots (right) for metabolites from SW1353 cells. (**A**) Uncompressed Monolayers (UM) vs. Uncompressed Microgels (UG). **(B**) Compressed Microgels (CG) vs. Compressed Monolayers (CM). (**C**) Uncompressed Monolayers vs. Compressed Monolayers. (**D**) Compressed Microgels vs. Uncompressed Microgels. All comparisons were made for cells cultured in complete media supplemented with 50 μg/mL L-sodium ascorbate. The numbers in the corners of the volcano plots denote the number of down- (left) and up- (right) regulated metabolites.

To examine the effects of microgel encapsulation on chondrocyte mechanotransduction, SW1353 cells were encapsulated in alginate microgels, and the microgels were suspended in a 4.5% *w*/*w* agarose solution, poured into a cylindrical mold, and gelled (Figure 1B). SW1353 cells are known to have differing gene expression from HPCs [36], but were chosen for this proof of concept study as they are easier to culture. Metabolomic profiling was performed on compressed and uncompressed constructs. Dynamic compression at 1.1 Hz was applied using a custom-built bioreactor for 15 min (Figure 1C) [8]. The sample groups included cells encapsulated in microgels before embedding in agarose and monolayer control cells encapsulated directly in agarose after monolayer culture. Thus, four conditions of SW1353 cells in agarose were compared: cells in microgels that were compressed and uncompressed, and cells from monolayers that were compressed and uncompressed as a control. Using heatmaps, principal components analysis, and volcano plots, distinct metabolic profiles between these four conditions were found using HPLC–MS (Figure 4 and Figure 5). Both hierarchical clustering and principal components analysis (PCA) found substantial differences between the metabolomic profiles of microgels and monolayer chondrocytes, independent of compression (Figure 4). Hierarchical clustering of the top 25 metabolites that differed between all groups showed a large cluster of metabolites that were downregulated in uncompressed microgels (Figure 4A, light-purple columns) and another cluster that was up-regulated in the compressed monolayer. (Figure 4A, bright-green columns).

PCA is an unsupervised method of assessing overall variation and potential similarity between samples among different experimental groups. The microgel and monolayer groups displayed clear separation when projected onto the principal components, indicating that these groups had distinct metabolomic profiles (Figure 4B, green monolayer compared to purple microgels). These groups also showed moderate overlap between the compressed and uncompressed samples, which was further analyzed using two-sample PCA analysis and volcano plots (Figure 5).

There were substantial differences between the metabolomic profiles of uncompressed microgel and uncompressed monolayer samples, as seen in the volcano plots, where the *x*-axis is the relative fold change in metabolite presence and the *y*-axis is the negative logarithm of the *p*-value for each metabolite. Significantly different metabolites are shown in pink, with 174 metabolites up-regulated in uncompressed microgels and 589 up-regulated in uncompressed monolayers (Figure 5A, right). Furthermore, there were substantial differences between the profiles of compressed microgels and compressed monolayer cells upon pairwise comparison (Figure 5B). The metabolomic profiles differed between the compressed microgels and compressed monolayers, with 75 metabolites up-regulated in microgels and 399 metabolites up-regulated in the monolayer samples (Figure 5B, right). Within both the monolayer and microgel groups, compression induced a robust metabolomic response for SW1353 chondrocytes (Figure 5C,D). Furthermore, the compressed samples showed decreased variability in the metabolomic profiles, as previously observed [8,22]. Dynamic compression in microgels induced the upregulation of 197 metabolites and downregulation of 13 metabolites when compared with uncompressed controls (Figure 5D, right). Finally, microgels had distinct compression-induced responses from the compressed monolayer controls (Figure 5B).

To identify the patterns of coregulated metabolites between groups, hierarchical clustering was applied to the median metabolite intensities. This analysis was performed across all eight experimental groups—microgels and monolayers cultured with and without ascorbate that were compressed and uncompressed—to capture all potential experimental features. This median clustering also found similarity between the uncompressed microgels cultured with ascorbate and compressed microgels without ascorbate, indicating that compression can mimic the effects of ascorbate on chondrocyte metabolomic profiles (Figures S3–S7).

To determine the biological relevance of coregulated metabolites, each cluster from the median heatmap (Figure S3) was assessed for cellular pathways. The resulting up-regulated pathways (false discovery rate-corrected *p*-values < 0.05) were from four main groups: compressed microgels cultured with ascorbate (Figure S5A Cluster #3), compressed monolayers cultured with ascorbate (Figure S7 Cluster #7), compressed monolayers cultured without ascorbate (Figures S4B and S5B Clusters #2 and 4), and uncompressed monolayers cultured without ascorbate (Figure S6B Cluster #6). The key pathway results are presented here with the complete results in the supplemental information (Figure S8 and Table S2).

We detected many pathways that were solely up-regulated in the compressed microgels cultured with ascorbate (Figure S5A Cluster #3). Here, the main pathways were associated with amino acid, energy, and hormone-related metabolism. Two vitamin B pathways were also significantly up-regulated—vitamin B6 and vitamin B5 metabolism. Vitamin B6 is involved in more than 140 different metabolic reactions in cells and, more importantly for this case, the supplementation of vitamin B6 reduces pro-inflammatory responses by suppressing pro-inflammatory cytokines IL-6 and TNF-alpha in patients with rheumatoid arthritis [37]. Meanwhile, vitamin B5 has the potential for maintaining bone homeostasis [38]. Vitamin B5 is required to synthesize coenzyme-A, an essential cofactor for fatty acid metabolism that is also a known regulator of chondrocyte maturation [39,40].

Interestingly, many pathways relating to the ECM and PCM were up-regulated in compressed microgels cultured with ascorbate. The chondrocyte ECM and PCM are primarily composed of collagen. Collagen contains many glycine and proline residues. The data showed that arginine and proline metabolism were up-regulated in compressed microgels, including proline upregulation. This pathway involves the biosynthesis and metabolism of several amino acids, including arginine, ornithine, proline, citrulline, and glutamate in mammals [41]. Additionally, N-glycan biosynthesis was up-regulated in compressed microgels cultured with ascorbate. Matrix proteoglycans utilize N-glycans and provide much of the compressive strength of cartilage by regulating matrix hydration through interactions with water. The compressed microgels cultured with ascorbate and the compressed monolayer cultured with ascorbate both share the pathways for glycine, serine, alanine, and threonine metabolism. These amino acids are also needed to synthesize PCM and ECM. Together, these pathways and the microscopy images suggest that collagen and matrix synthesis were likely up-regulated in the compressed microgels cultured with ascorbate. Furthermore, both the compression and culture conditions play a role in ECM and PCM production, but further mechanistic studies are necessary to determine how these key environmental stimuli drive matrix production.

Other common pathways between the compressed microgel and monolayer samples with ascorbate are butanoate metabolism and glutathione metabolism. The former is responsible for L-glutamate degradation, followed by a series of reactions that produce CoA-conjugated compounds. The latter is considered a major antioxidant and one of the indicators of cellular oxidative stress. While both groups showed the same metabolite again (pyroglutamic acid), the monolayer compressed group expressed trypanothione compared to the microgel compressed group, which expressed gamma-glutamylcysteine.

In the compressed monolayers cultured with ascorbate (Figure S7 Cluster #7), the upregulation of many CoA-conjugated metabolites was detected, including Palmityl-CoA, Linoleoyl-CoA, and Arachidonyl-CoA. Importantly, Acetyl-CoA is a downstream product of these Acyl-CoA metabolites and regulates chondrocyte maturation [40]. Acetyl-CoA provides acetyl groups to the citric acid cycle, a key component of energy metabolism. Energy pathways also play an important role in the development, maintenance, and repair of the extracellular matrix (ECM) [42]. These results further confirm previous studies, which found that compression is a key factor for modeling chondrocyte mechanotransduction [9].

We note that metabolomic profiling focused on cellular metabolites, while the profiles of metabolites and other molecules secreted into the media remain unknown. Additionally, studies on primary cells involved passaging in a monolayer in normoxia, which may affect the chondrocyte phenotype [43]. Due to low HPC availability, the bulk of the metabolic studies were performed in SW1353 chondrosarcoma cells to optimize the experimental system. Nonetheless, collagen formation observed around HPC in the alginate microgels (Figure 2A–C) yields promising data for future metabolomic studies using HPCs that will provide exciting advances in single-cell chondrocyte microgels to advance osteoarthritis and tissue engineering.

## 4. Conclusions

Chondrocytes were encapsulated in alginate microgels, imaged over ten days, and evaluated for collagen formation. These data indicate that collagen VI production was greatly increased in microgels compared to monolayer cultures. The HPCs produced more collagen VI than the SW1353 cells when cultured in microgels or monolayers, as observed using immunofluorescence with confocal microscopy. SW1353 cells cultured in microgels produced more collagen VI than SW1353 cells cultured in monolayer using immunofluorescence with confocal microscopy and auto-fluorescence using epifluorescence microscopy. Thus, these results indicate that chondrocytes are more likely to form a PCM comprised of collagen VI in 3-D alginate microgels than in monolayers. Additionally, chondrocytes were encapsulated in physiologically stiff agarose gels and subjected to physiological dynamic compression. The cells cultured in microgels had substantially different metabolomic profiles compared to the cells cultured in monolayers. Compression induced a robust metabolic response in both the monolayer controls and microgel-encapsulated chondrocytes. Upon metabolomic pathway analysis, the microgel-encapsulated chondrocytes were found to have up-regulated pathways related to amino acid synthesis and central energy metabolism. Together, these pathways and the microscopy images suggest that collagen and matrix synthesis is increased in microgels compared to monolayers.

In summary, we show that microgels created using drop-based microfluidics can provide a robust 3-D culture environment for individual chondrons *in vitro*. The encapsulation of the cells in small volumes of alginate enables the development of collagen VI-rich PCM surrounding individual cells. This novel method eliminates the use of potentially harmful digestive processes that are typically applied to isolate single cells in traditional bulk alginate cultures in order to isolate cells with developed PCM. We demonstrate that these single-cell microgels can be embedded in physiologically stiff agarose for studying the metabolic response to compression. This is the first instance of chondrons encapsulated in alginate microgels and embedded in physiologically stiff agarose, enabling more realistic *in vitro* models of chondrocyte mechanotransduction that better mimic the *in vivo* structure. These microscale constructs allow the manipulation of thousands of single cells in microenvironments that can be embedded into other hydrogels of interest. The mechanotransduction techniques developed here will be applied towards future studies of HPCs, known to have different gene expression patterns compared to SW1353 cells [36]. The microscale control offered by drop-based encapsulation should enable better manipulation of the highly limited number of HPCs that are isolated from cartilage [2], as well as the exploration of growth conditions for the optimal cell viability of these valuable cells. We expect that our work will have a great impact on future studies on chondrocyte mechanotransduction and tissue engineering.

## Data Availability

The data presented in this study are available as supplementary files and on request from the corresponding author.

## Supplementary Materials

The following supporting information can be downloaded at: https://www.mdpi.com/article/10.3390/cells11050900/s1, Figure S1: Brightfield and fluorescence images of SW1353 cells; Figure S2: Storage and loss moduli versus angular frequency of hydrogels; Figure S3: Median metabolites across all experimental groups; Figure S4: Median metabolites across all experimental group for clusters 1 and 2; Figure S5: Median metabolites across all experimental group for clusters 3 and 4; Figure S6: Median metabolites across all experimental group for clusters 5 and 6; Figure S7: Median metabolites across all experimental group for cluster 7; Figure S8: Venn diagram of significant pathways for each group; Table S1: Storage and loss moduli versus angular frequency of hydrogels; Table S2: All significant pathways found and the corresponding gamma values and matched metabolites.

## Abbreviations

3-D	Three-dimensional
*w*/*w*	Weight per weight
IRB	Institutional review board
*v*/*v*	Volume per volume
I.U.	International Unit
*Q*	Volumetric Flowrate
