# Supplementary material for: Metabolomic Profiling and Mechanotransduction of Single Chondrocytes Encapsulated in Alginate Microgels
Source: Cells. 2022 Mar 5;11(5):900. doi: 10.3390/cells11050900 (PMC8909502; doi:10.3390/cells11050900)
Supplement: Supplementary file 1 [file cells-11-00900-s001.zip › cells-1577651-SI.pdf]

Supporting Information

Metabolomic Profiling and Mechanotransduction of Single Chondrocytes Encapsulated in Alginate Microgels

Authors: Jacob P. Fredrikson, Priyanka P. Brahmachary, Ayten E. Erdoğan, Zachary K. Archambault, James N. Wilking, Ronald K. June, Connie B. Chang**

Number of Supplemental Pages: 18

Number of Supplemental Figures: 8

Number of Supplemental Tables: 2

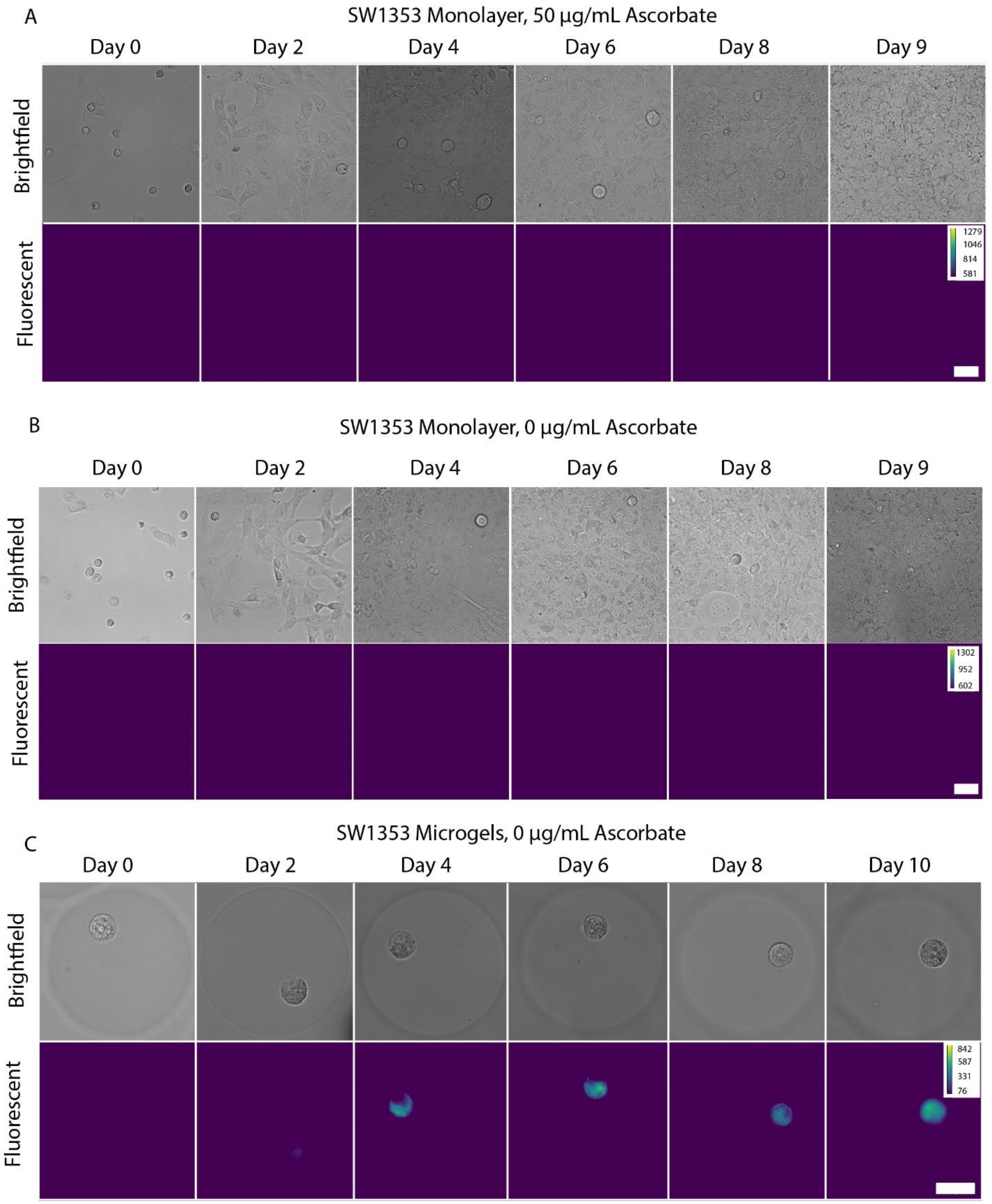

Figure S1. Brightfield and fluorescence images of microgels without and with ascorbate of (A) SW1353 cells grown on monolayers with 50 µg mL^{-1} ascorbate. Scale bar = 50 µm (B) SW1353 cells grown on monolayers with 0 µg mL^{-1} ascorbate. Scale bar = 50 µm (C) SW1353 cells encapsulated in alginate microgels and cultured with 0 µg mL^{-1} ascorbate Scale bar =25 µm

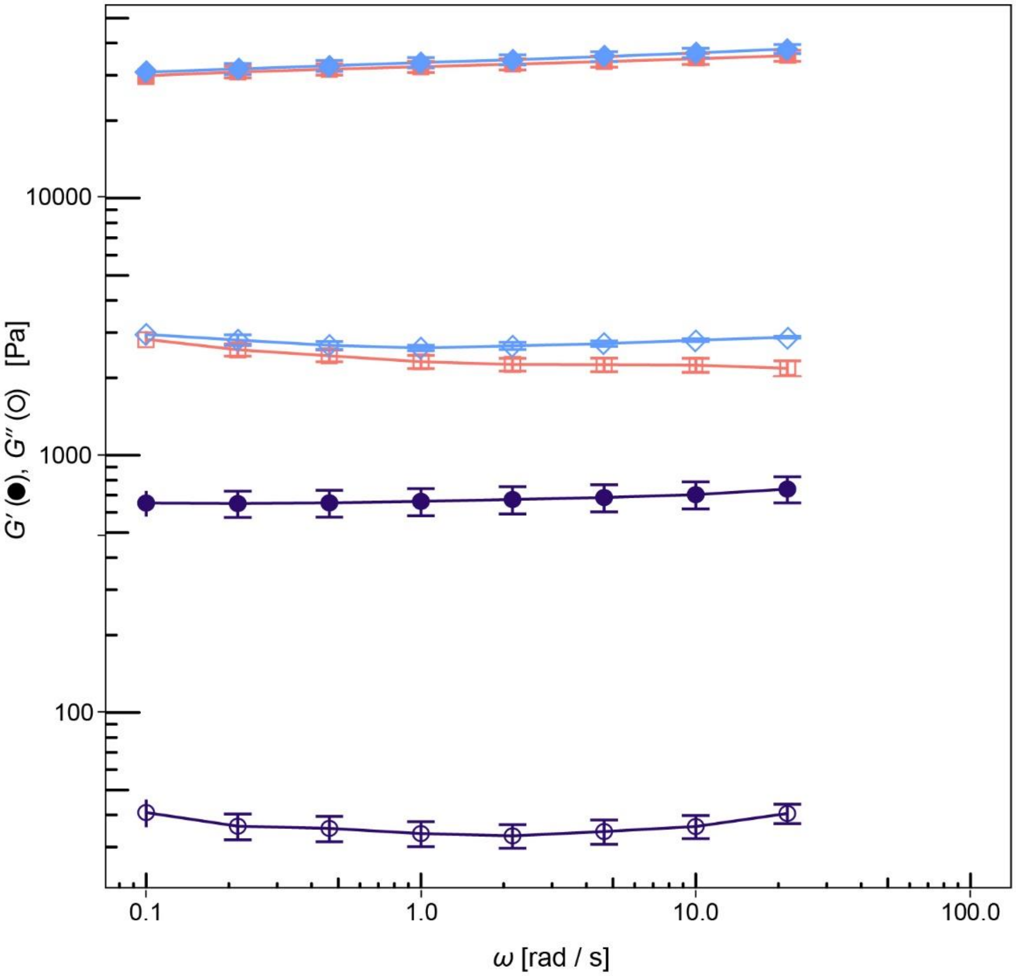

Figure S2. Storage (*G',* closed symbols) and loss (*G'',* open symbols) moduli versus angular frequency, T = 25 °C, for 4.5% *w/w* agarose (squares), 1.5% *w/w* alginate (circles), and 4.5% *w/w* agarose with 20% *v/v* 1.5% *w/w* alginate microgels (diamonds).

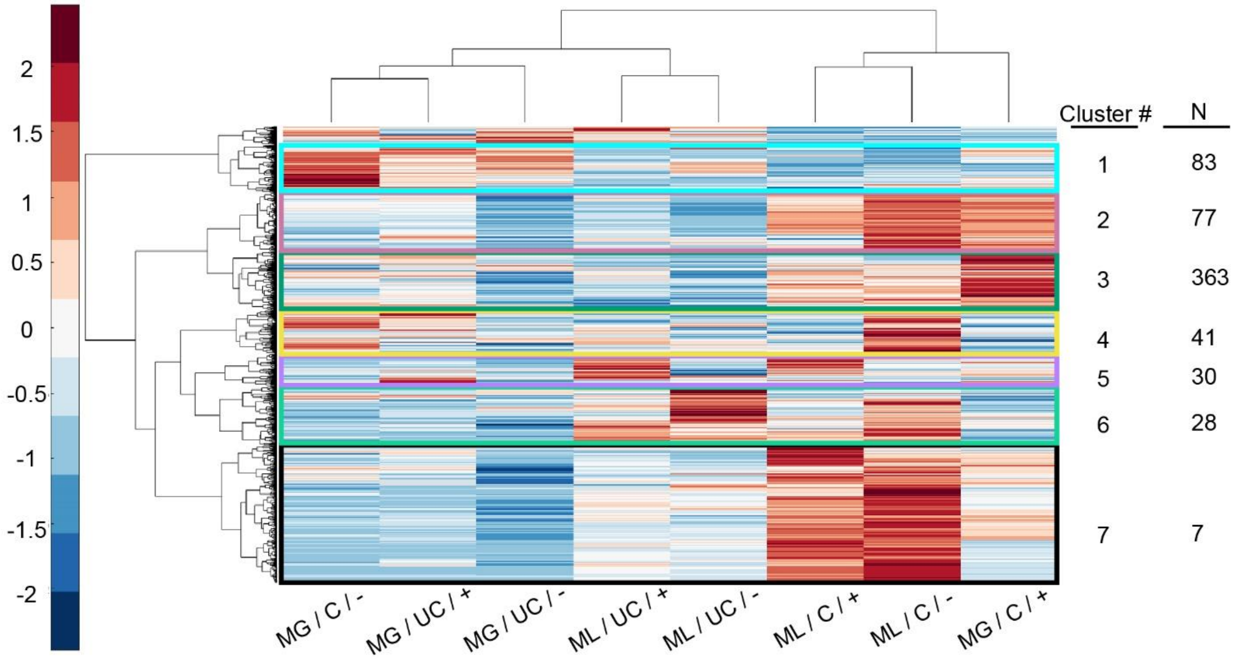

Figure S3. Median metabolites across all experimental groups. Metabolite clusters (colored boxes) were subjected to pathway enrichment analysis, but no significant pathways were found after false discovery rate correction. ML (Monolayer), MG (Microgel), C (Compressed), UC (Uncompressed), + (complete media supplemented with 50 μg mL^{-1} L-sodium ascorbate), - (complete media lacking L-sodium ascorbate).

A

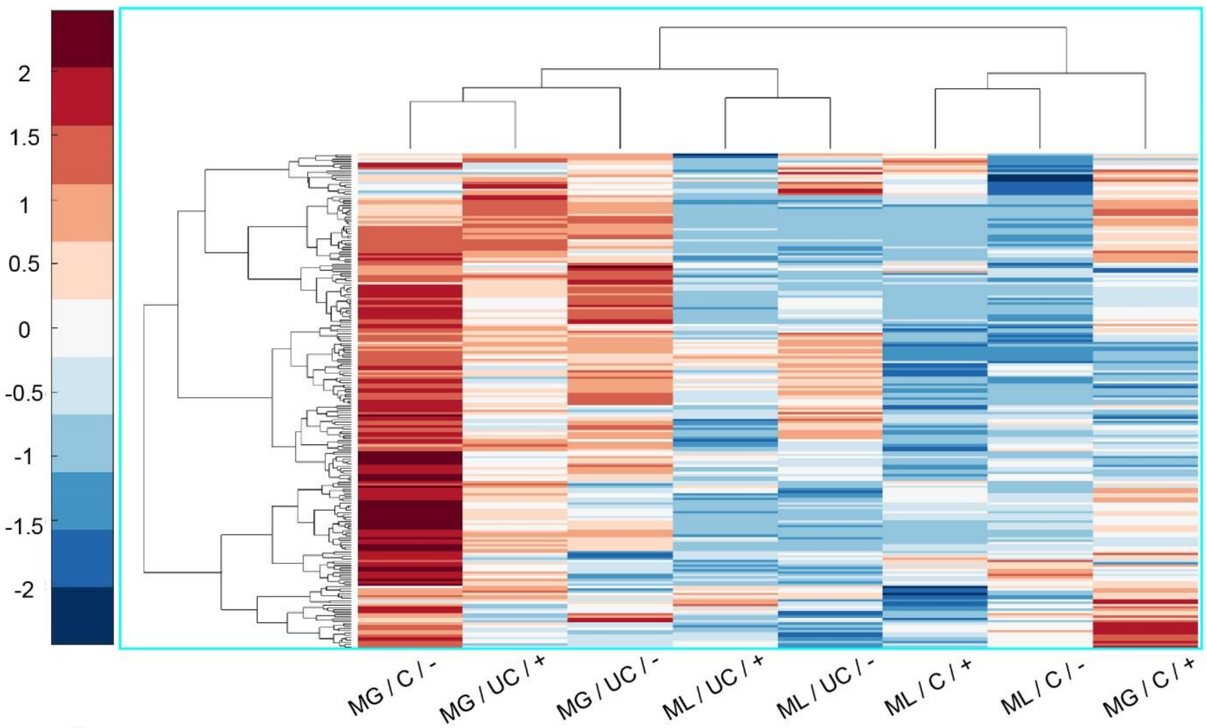
2
1.5
1
0.5
0
-0.5
-1
-1.5
-2
MG / C / -
MG / UC / +
MG / UC / -
ML / UC / +
ML / UC / -
ML / C / +
ML / C / -
MG / C / +

B

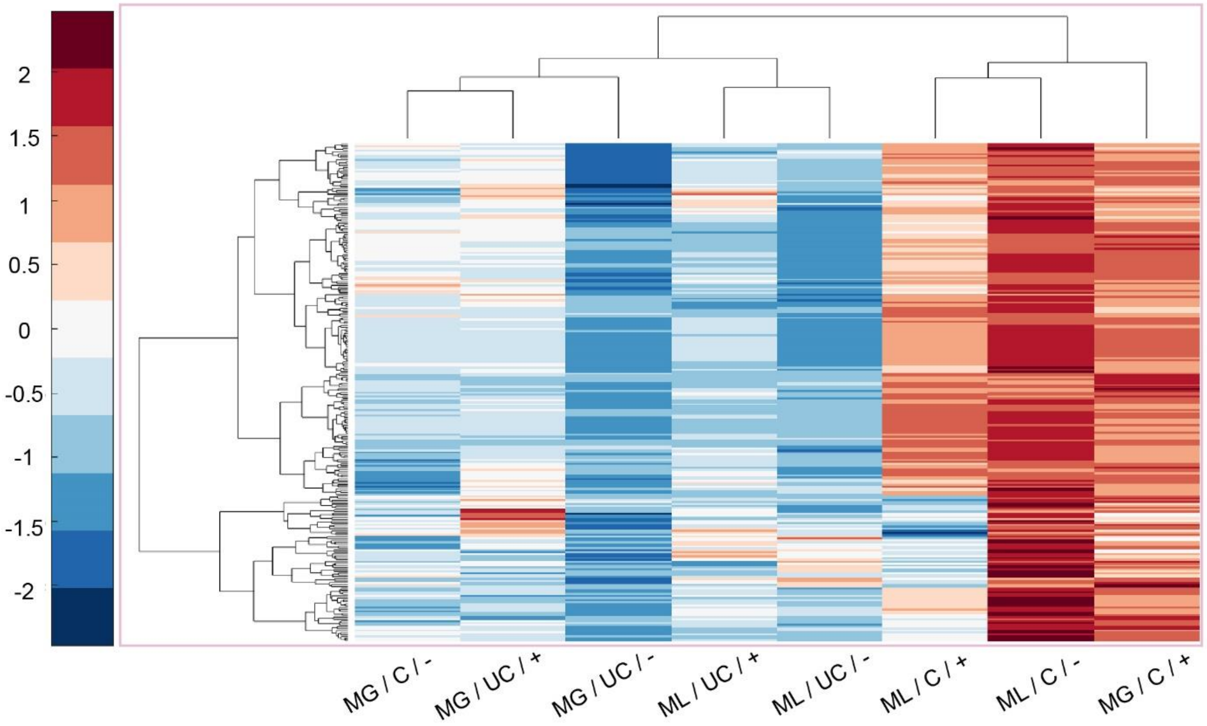
2
1.5
1
0.5
0
-0.5
-1
-1.5
-2
MG / C / -
MG / UC / +
MG / UC / -
ML / UC / +
ML / UC / -
ML / C / +
ML / C / -
MG / C / +

Figure S4. Median metabolites across all experimental group. (A) Metabolite cluster 1 and (B) cluster 2 from SI figure 4 were further subjected to pathway enrichment analysis, but no significant pathways were found after false discovery rate correction. ML (Monolayer), MG (Microgel), C (Compressed), UC (Uncompressed), + (complete media supplemented with 50 μg mL^{-1} L-sodium ascorbate), - (complete media lacking L-sodium ascorbate).

A

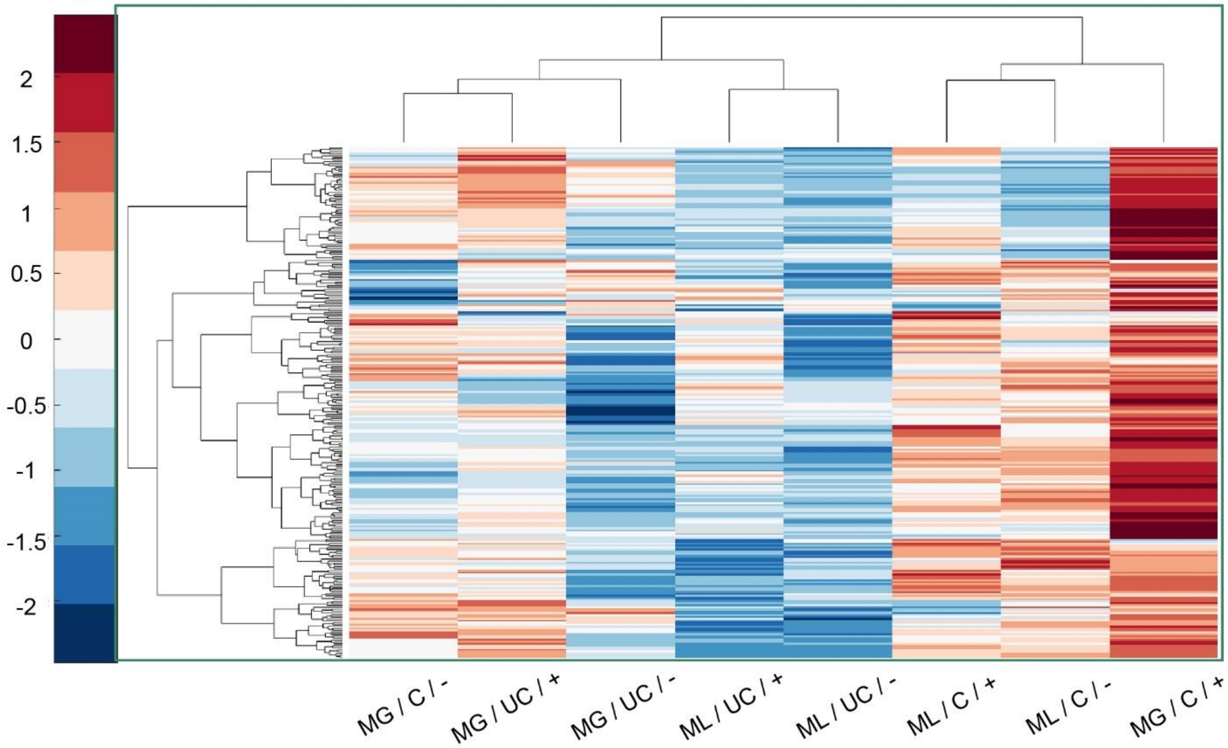
2
1.5
1
0.5
0
-0.5
-1
-1.5
-2
MG / C / -
MG / UC / +
MG / UC / -
ML / UC / +
ML / UC / -
ML / C / +
ML / C / -
MG / C / +

B

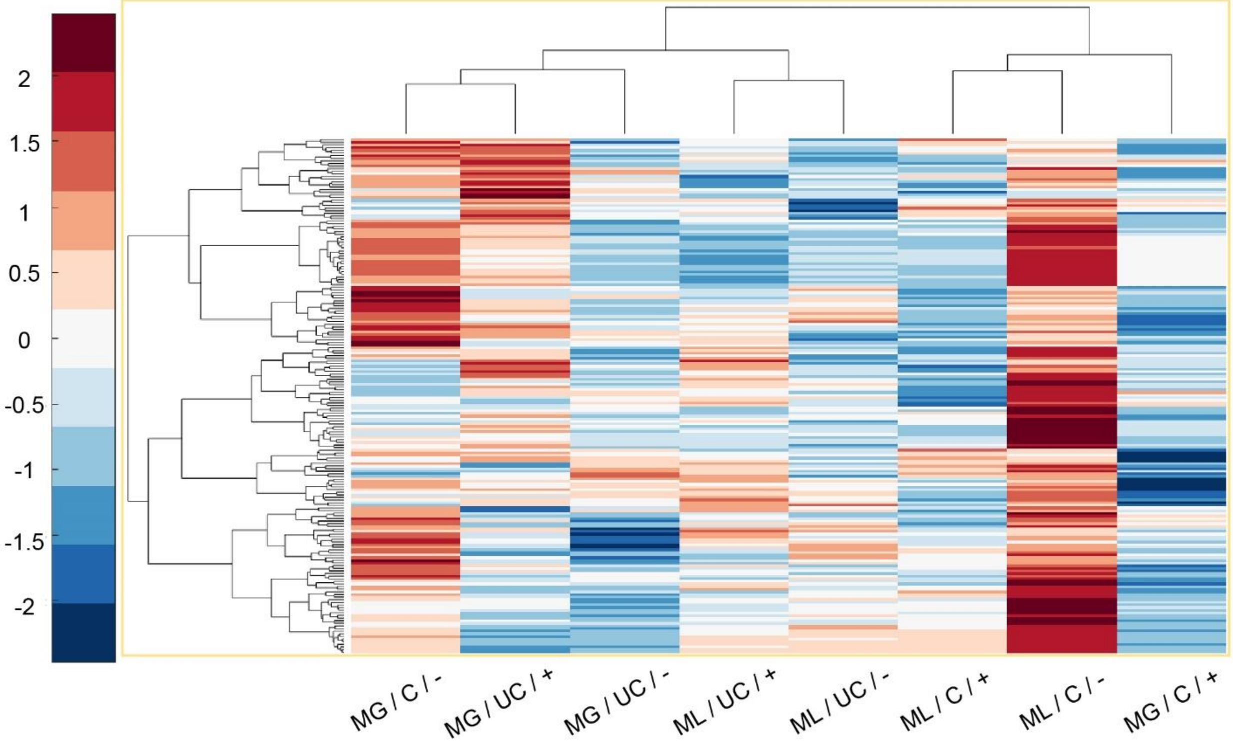
2
1.5
1
0.5
0
-0.5
-1
-1.5
-2
MG / C / -
MG / UC / +
MG / UC / -
ML / UC / +
ML / UC / -
ML / C / +
ML / C / -
MG / C / +

Figure S5. Median metabolites across all experimental group. (A) Metabolite cluster 3 and (B) cluster 4 from SI Figure 4 were subjected to pathway enrichment analysis, but no significant pathways were found after false discovery rate correction. ML (Monolayer), MG (Microgel), C (Compressed), UC (Uncompressed), + (complete media supplemented with 50 μg mL^{-1} L-sodium ascorbate), - (complete media lacking L-sodium ascorbate).

A

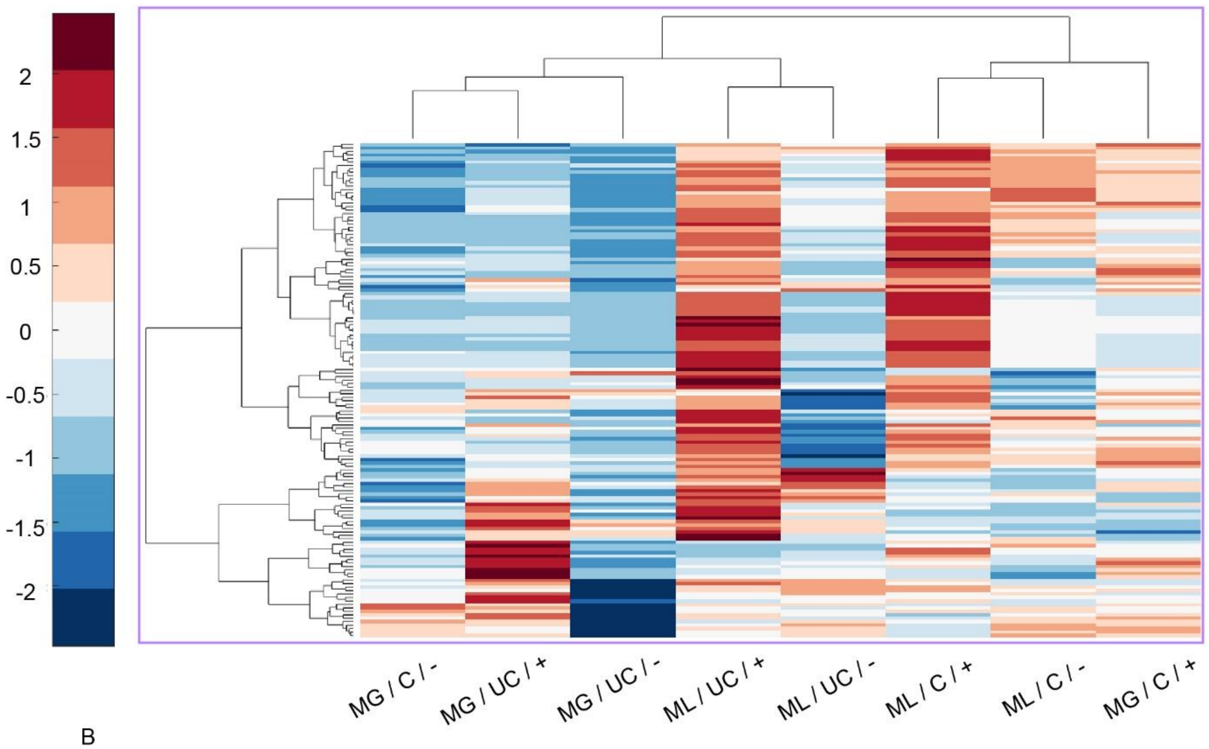
2
1.5
1
0.5
0
-0.5
-1
-1.5
-2
MG / C / -
MG / UC / +
MG / UC / -
ML / UC / +
ML / UC / -
ML / C / +
ML / C / -
MG / C / +

B

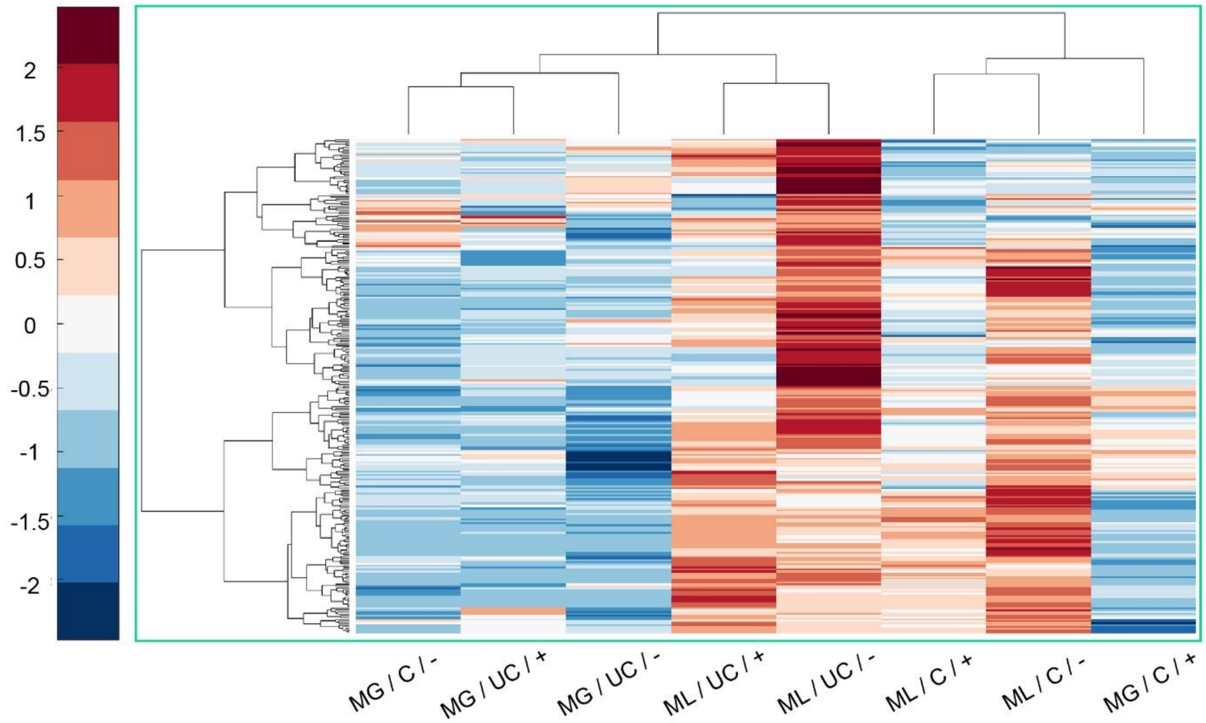
2
1.5
1
0.5
0
-0.5
-1
-1.5
-2
MG / C / -
MG / UC / +
MG / UC / -
ML / UC / +
ML / UC / -
ML / C / +
ML / C / -
MG / C / +

Figure S6. Median metabolites across all experimental group. (A) Metabolite cluster 5 and (B) cluster 6 from SI Figure 4 were subjected to pathway enrichment analysis, but no significant pathways were found after false discovery rate correction. ML (Monolayer), MG (Microgel), C (Compressed), UC (Uncompressed), + (complete media supplemented with 50 µg mL^{-1} L-sodium ascorbate), - (complete media lacking L-sodium ascorbate).

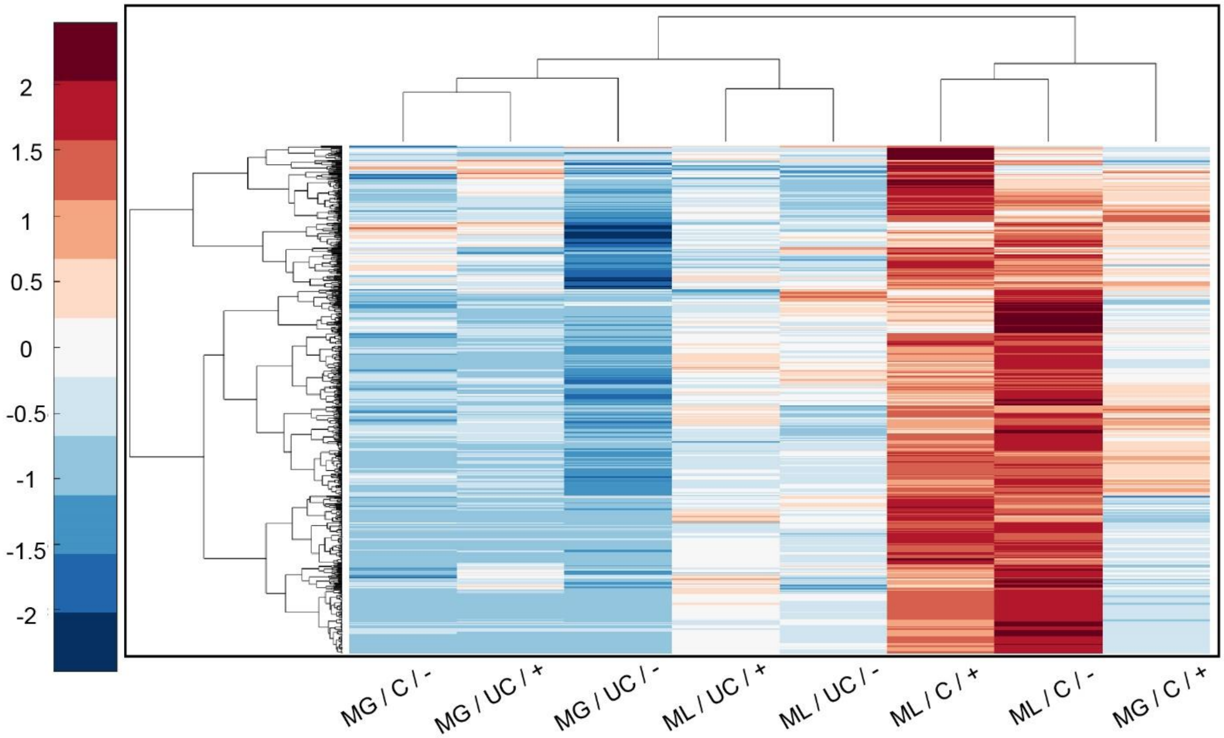

Figure S7. Median metabolites across all experimental group. Metabolite cluster 7 from SI Figure 4 was subjected to pathway enrichment analysis, but no significant pathways were found after false discovery rate correction. ML (Monolayer), MG (Microgel), C (Compressed), UC (Uncompressed), + (complete media supplemented with 50 µg mL^{-1} L-sodium ascorbate), - (complete media lacking L-sodium ascorbate).

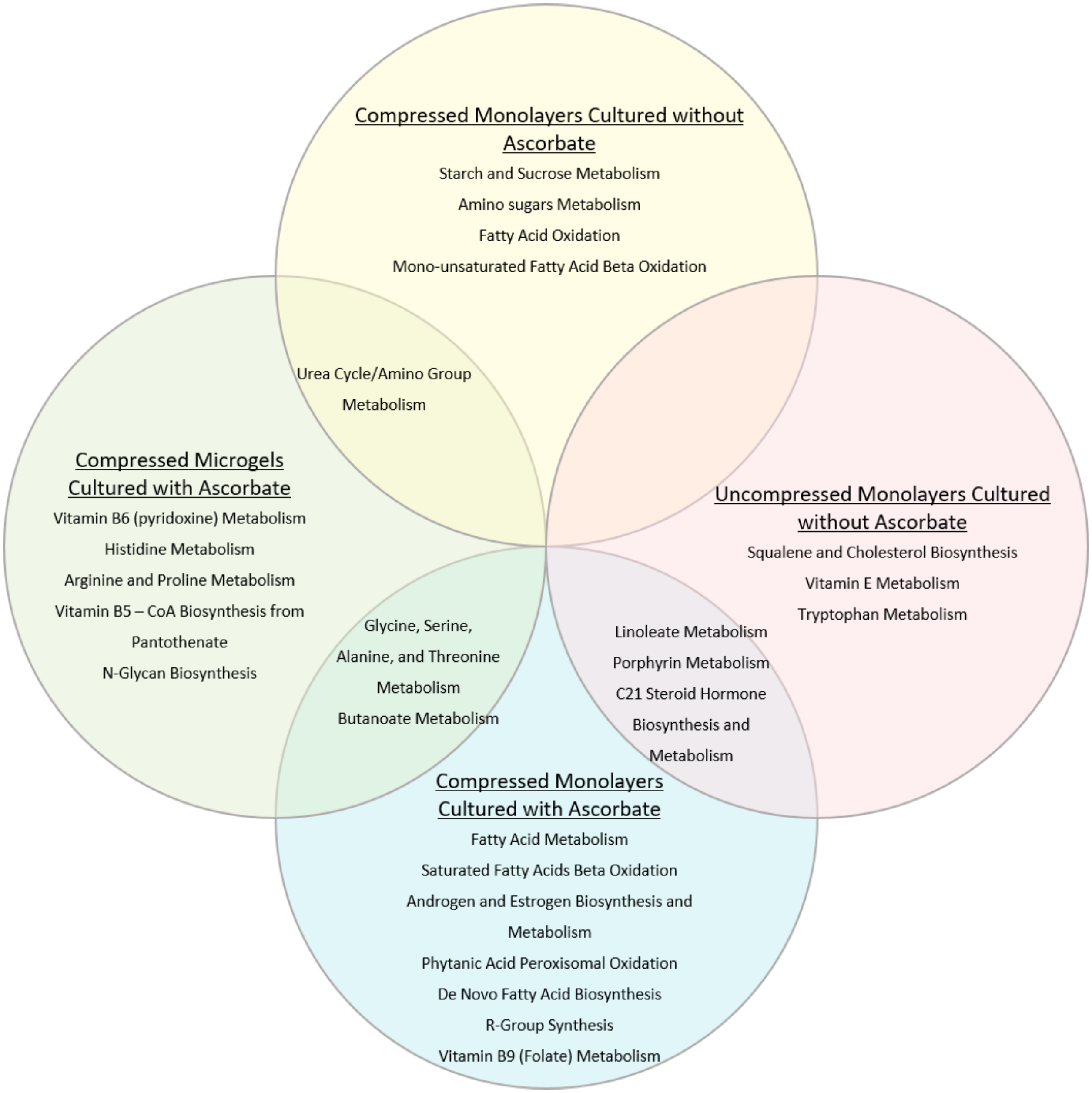

Figure S8. Venn diagram of significant pathways for each group.

Hydrogel	ω [rad/s]	G' [Pa]			G'' [Pa]		
4.5% *w/w* Agarose	0.100	3.0E+04	±	2.9E+03	2.8E+03	±	2.4E+02
	0.215	3.1E+04	±	2.7E+03	2.6E+03	±	2.4E+02
	0.464	3.2E+04	±	2.8E+03	2.4E+03	±	2.2E+02
	1.000	3.2E+04	±	2.8E+03	2.3E+03	±	2.3E+02
	2.154	3.3E+04	±	2.8E+03	2.3E+03	±	2.2E+02
	4.642	3.4E+04	±	2.9E+03	2.2E+03	±	2.4E+02
	10.000	3.5E+04	±	3.0E+03	2.2E+03	±	2.4E+02
	21.544	3.6E+04	±	3.0E+03	2.2E+03	±	2.6E+02
1.5 % *w/w* Alginate	0.100	6.5E+02	±	1.8E+02	4.1E+01	±	1.2E+01
	0.215	6.5E+02	±	1.9E+02	3.6E+01	±	1.0E+01
	0.464	6.5E+02	±	1.9E+02	3.5E+01	±	9.8E+00
	1.000	6.6E+02	±	2.0E+02	3.4E+01	±	9.3E+00
	2.154	6.7E+02	±	2.0E+02	3.3E+01	±	8.5E+00
	4.642	6.9E+02	±	2.0E+02	3.4E+01	±	9.2E+00
	10.000	7.0E+02	±	2.1E+02	3.6E+01	±	9.1E+00
	21.544	7.4E+02	±	2.1E+02	4.1E+01	±	8.6E+00
Agarose + 20% *v/v* Alginate Microgels	0.100	3.1E+04	±	2.7E+03	2.9E+03	±	1.6E+02
	0.215	3.2E+04	±	2.8E+03	2.8E+03	±	2.3E+02
	0.464	3.3E+04	±	2.7E+03	2.7E+03	±	1.6E+02
	1.000	3.4E+04	±	2.7E+03	2.6E+03	±	1.0E+02
	2.154	3.4E+04	±	2.7E+03	2.7E+03	±	1.5E+02
	4.642	3.5E+04	±	2.7E+03	2.7E+03	±	1.1E+02
	10.000	3.7E+04	±	2.7E+03	2.8E+03	±	5.9E+01
	21.544	3.8E+04	±	2.7E+03	2.9E+03	±	4.2E+01

Table S1. Elastic (*G*') and Storage (*G''*) moduli of 4.5% *w/w* agarose, 1.5% *w/w* alginate, and 4.5% *w/w* agarose with 20% *v/v* 1.5% *w/w* alginate microgels for varying angular frequencies. Rheology was performed on a TA Instruments AR-G2 rheometer with a parallel plate geometry.

Cluster #2 – Compressed Monolayers Cultured without Ascorbate (Up-regulated)

	Gamma	Matched Metabolites
Starch and Sucrose Metabolism	0.0441	*Maltopentaose<br>*D-Glucose<br>*D-Glucose<br>*Alpha-D-Glucose
Urea cycle/amino group metabolism	0.048281	*Creatinine<br>*L-Arginine<br>*2-(3-Carboxy-3-(methylammonio)propyl)-L-histidine<br>*L-Methionine – the 1st amino group (essential)<br>*Creatine
Aminosugars metabolism	0.048739	*Chitin<br>*(N-acetylneuraminosyl(a2-6)lactosamine)<br>*Phosphoenolpyruvic acid

Cluster #3 – Compressed Microgels Cultured with Ascorbate (Up-regulated)

	Gamma	Matched Metabolites
Vitamin B6 (pyridoxine) metabolism	0.035844	*Pyridoxamine<br>*Pyridoxine
Urea cycle/amino group metabolism	0.036024	*L-Proline<br>*Hippuric acid<br>*Citrulline<br>*4-Acetamidobutanoic acid<br>*4-Aminobutyraldehyde<br>*N-Methylphenylethanolamine

		*Norepinephrine
Histidine metabolism	0.036669	*5-Formiminotetrahydrofolic acid<br>*Methylimidazoleacetic acid<br>*Carnosine
Glycine, serine, alanine and threonine metabolism	0.040908	*Betaine<br>*2-methylbutyrylglycine<br>*Isovalerylglycine<br>*L-Allothreonine<br>*L-Threonine
Arginine and Proline Metabolism	0.040908	*L-Proline<br>*Citrulline<br>*4-Acetamidobutanoic acid<br>*1-Pyrroline-2-carboxylic acid<br>*4-Aminobutyraldehyde<br>*D-Proline<br>*Pyrroline hydroxycarboxylic acid
Vitamin B5 - CoA biosynthesis from pantothenate	0.045044	*4-Phosphopantothenoylcysteine<br>*Pantetheine
Butanoate metabolism	0.045044	*2-Keto-6-acetamidocaproate<br>*5-Aminopentanoic acid
Glutathione Metabolism	0.045044	*Pyroglutamic acid<br>*gamma-Glutamylcysteine
N-Glycan biosynthesis	0.04861	*Uridine diphosphategalactose<br>*Uridine diphosphate glucose<br>*N,N'-Diacetylchitobiosyldiphosphodolichol<br>*Dolichol-20

Cluster #4 - Compressed Monolayers Cultured without Ascorbate (Up-regulated)

	Gamma	Matched Metabolites

Fatty acid oxidation	0.041484	*2,6 dimethylheptanoyl-CoA<br>*Gamma-linolenoyl-CoA<br>*4,8 dimethylnonanoyl-CoA<br>*Trans-Octadec-2-enoyl-CoA<br>* Hexadecenoyl-CoA<br>* Vaccenyl coenzyme A<br>*Octadecenoyl-CoA
Mono-unsaturated fatty acid beta-oxidation	0.047503	* 3-oxolaur-cis-5-enoyl-CoA<br>*Palmitoleoyl-CoA

Cluster #6 - Uncompressed Monolayers Cultured without Ascorbate (Up-regulated)

	Gamma	Matched Metabolites
Linoleate metabolism	0.033754	*LysoPC(18:1(9Z))<br>*(11S)-11-Hydroperoxylinoleic acid<br>* 9,10-12,13-diepoxy-octadecanoate
Squalene and cholesterol biosynthesis	0.041083	* 4,4-dimethylcholesta-8(9),14-dien-3beta-ol<br>*Presqualene diphosphate<br>*4,4-Dimethyl-5a-cholesta-8,24-dien-3-b-ol<br>*Avenasterol<br>* Delta7-Avenasterol
Porphyrin metabolism	0.04495	*Protoporphyrinogen IX<br>*Uroporphyrin I<br>*Uroporphyrin III
C21-steroid hormone biosynthesis and metabolism	0.04495	*Cholesterol sulfate<br>*Estrone sulfate

Vitamin E metabolism	0.048938	*Phenylacetaldehyde<br>*9'-carboxy-alpha-chromanol
Tryptophan metabolism	0.052097	*Cyclic 3-hydroxymelatonin<br>*6-Hydroxymelatonin<br>*Melatonin radical<br>*Tetrahydrobiopterin

Cluster #7 – Compressed Monolayers Cultured with and without Ascorbate (Up-regulated)

	Gamma	Matched Metabolites
Fatty Acid Metabolism	0.011922	*Tetradecanoyl-CoA<br>*Palmityl-CoA<br>*(2E)-Dodecenoyl-CoA<br>*3-Oxotetradecanoyl-CoA<br>*3-dodecenoyl CoA
Saturated fatty acids beta-oxidation	0.011922	*Tetradecanoyl-CoA<br>*Palmityl-CoA<br>*(S)-3-Hydroxytetradecanoyl-CoA<br>*3-Oxotetradecanoyl-CoA<br>*(S)-3-Hydroxydodecanoyl-CoA<br>*(2E)-Dodecenoyl-CoA
Androgen and estrogen biosynthesis and metabolism	0.017094	*Estrone sulfate<br>*Trypanothione disulfide<br>*17-beta-Estradiol-3-glucuronide<br>*Androstenedione<br>*Estrone glucuronide
Porphyrin metabolism	0.018407	*Heme O<br>*Coproporphyrin I<br>*Protoporphyrinogen IX<br>*Coproporphyrin III

C21-steroid hormone biosynthesis and metabolism	0.018407	*Androst-4-ene-3,17-dione<br>*Estrone-glucuronide<br>*Estrone sulfate<br>*Androstenedione<br>*17beta-estradiol 3-glucosiduronic acid
Phytanic acid peroxisomal oxidation	0.021904	*Pristanoyl-CoA<br>*4,8,12-Trimethyltridecanoyl-CoA<br>*3(S)-2-hydroxyphytanoyl-CoA<br>*(2R)-pristanoyl-CoA;<br>*(2S)-pristanoyl-CoA<br>*3(R)-hydroxy-pristanoyl-CoA
De novo fatty acid biosynthesis	0.023437	*Palmityl-CoA<br>*Linoleoyl-CoA<br>*Tetradecanoyl-CoA;<br>*Arachidonyl-CoA<br>*3-hydroxyoctadecanoyl-CoA<br>*3-hydroxytetracosanoyl-CoA<br>*3-hydroxyeicosanoyl-CoA<br>*3(S)-hydroxy-11-cis-eicosenoyl-CoA<br>*Trans,cis-2,11-eicosadienoyl-CoA
Glycine, serine, alanine and threonine metabolism	0.031029	*Arachidonyl-CoA<br>*Creatine<br>*L-Allothreonine<br>*L-Threonine
R Group Synthesis	0.033308	*Palmityl-CoA<br>*Tetradecanoyl-CoA
Butanoate metabolism	0.033308	*2-Keto-6-acetamidocaproate<br>*5-Acetamidovalerate
Glutathione Metabolism	0.033308	*Pyroglutamic acid<br>*Trypanothione disulfide

Vitamin B9 (folate) metabolism	0.044339	*10-formyltetrahydrofolate-[Glu](5)<br>*Tetrahydrofolyl-[Glu](n)

Table S2. All significant pathways found and the corresponding gamma values and matched metabolites.
